# See me and hear me: a Photovoice study of Hispanic adolescents’ mental health conceptualization and priorities in the USA

**DOI:** 10.1093/heapro/daag032

**Published:** 2026-02-27

**Authors:** Carolina Vélez-Grau, Meghan Romanelli, Krystel Francis, Adriana Rios, Peter Lopez, Mswati Hanks, María Pineros-Leano

**Affiliations:** Boston College School of Social Work, 140 Commonwealth, Chestnut Hill, MA 02467, United States; University of Washington School of Social Work, 4101 15th Ave NE, Seattle, WA 98105, United States; Boston College School of Social Work, 140 Commonwealth, Chestnut Hill, MA 02467, United States; Boston College School of Social Work, 140 Commonwealth, Chestnut Hill, MA 02467, United States; Basement Trybe, 34 Moultrie Street, Boston, MA 02124, United States; Turn It Around Youth Enrichment Program, Massachusetts General Hospital Center for Community Health Improvement, 15 Green St, Charlestown, MA 02129, United States; Boston College School of Social Work, 140 Commonwealth, Chestnut Hill, MA 02467, United States

**Keywords:** Photovoice, Hispanic adolescents, mental health definition, CBPR

## Abstract

Hispanic adolescents experience disproportionately high rates of mental health issues and face systemic barriers to accessing care, yet their perspectives remain underrepresented in research. Understanding how they define mental health is critical, as it shapes beliefs, priorities, and help-seeking behaviors. This study employed Photovoice, a community-based participatory research (CBPR) method, to explore Hispanic adolescents’ conceptualizations of mental health and their priorities. Twelve adolescents (ages 13–17) from two youth centers in Greater Boston were divided into three groups. Each group participated in three meetings (a preparatory workshop, a focus group, and a feedback workshop) between June 2024 and February 2025. Participants took photographs reflecting their views on mental health, followed by reflective discussions using the SHOWeD technique. Reflexive thematic analysis was used to analyze data. Mental health was defined as the ability to feel, express, and manage emotions. Adolescents’ mental health priorities were (i) self-expression and coping with distress; (ii) the role of immigrant families in adolescent mental health; (iii) the influence of peers, trusted adults, and safe community spaces in adolescent mental health; and (iv) mental health stigma. Participants emphasized creative outlets and open dialogue, while identifying cultural expectations and intergenerational silence as barriers to emotional well-being. Results underscore the need for culturally responsive mental health promotion that centers adolescent voices. Interventions should foster safe spaces for expression, validate lived experiences, and address stigma within immigrant families and communities. Intervention strategies must be multisystemic and multilayered—including family and community settings—to advance mental health equity and ensure sustainable support for Hispanic adolescents.

Contribution to Health Promotion

- Photovoice empowered Hispanic adolescents to define mental health in their own words, addressing gaps in culturally responsive, adolescent-centered research.
- Adolescents identified cultural expectations, immigrant family dynamics, and intergenerational silence as key barriers to emotional well-being and support.
- Trusted adults and safe community spaces were seen as crucial supports for emotional expression and well-being.
- There is a need for developmentally and culturally responsive multisystemic approaches that validate lived experiences and address stigma in families, schools, and communities.

## Introduction

Hispanic adolescents, who comprise 25% of the US population under 18, experience high rates of poor mental health, sadness, and hopelessness—key indicators of depression—while simultaneously facing significant barriers to accessing mental health services (Mikolajczyk *et al*. 2007, Merikangas *et al*. 2010, Alegria *et al*. 2011, Centers for Disease Control and Prevention CDC 2024). Recent national data indicate that nearly one-third of Hispanic males and over half of Hispanic females ages 12–17 experience symptoms of depression (Verlenden *et al*. 2024). These disparities were exacerbated by the COVID-19 pandemic, which introduced unique stressors for Hispanic adolescents, including heightened discrimination, emotional, and behavioral difficulties, disruptions in family cohesion, and academic instability (Roche *et al*. 2022, Polo *et al*. 2024, Sanchez *et al*. 2024, Vélez-Grau *et al*. 2024).

Despite the heightened need for mental health services, Hispanic adolescents remain critically underserved and face numerous barriers to care. Stigma surrounding mental health remains an ongoing obstacle (Leal 2005, Grieb *et al*. 2023) alongside fears of burdening family members (Martinez 2017, Vélez-Grau *et al*. 2024), concerns about confidentiality breaches (Ijadi-Maghsoodi *et al*. 2018), and limited access to culturally competent providers (Tran and Tran 2022). Collectively, these connected challenges point to an urgent need for culturally responsive mental health interventions that address both the systemic and relational contexts shaping adolescent well-being. Yet, without directly engaging adolescents themselves, there is a risk of designing services that fail to reflect their lived realities, priorities, and definitions of mental health. This disconnect can further widen the gap between mental health needs and service utilization.

### Theoretical framework

Adolescence is a critical developmental period marked by emotional, cognitive, and social changes. During this time, mental health becomes a central component of overall well-being. According to the Integrative Risk and Resilience Model for Immigrant-Origin Children (Suárez-Orozco *et al*. 2018), children and adolescent development is molded by multiple interacting contexts, including family, culture, and sociopolitical environments. For the majority of Hispanic adolescents who live in immigrant-origin households, mental well-being is a balance between risks, such as ethnic discrimination and poverty, and resilience factors like cultural identity and strong family support. Understanding how adolescents navigate these “multi-layered dynamics” is essential for creating ecological interventions that address more than just individual-level symptoms (Suárez-Orozco *et al*. 2018).

### Photovoice in mental health research with adolescents

Traditionally, adolescent mental health research has utilized adult-centered perspectives to define youth needs and inform interventions (Zaff *et al*. 2016). While these approaches have contributed valuable information, they often overlook adolescents’ lived experiences and contextual nuances that shape their mental health (Yonas *et al*. 2013). Specifically, some studies have found that adolescent narratives provide a level of complexity that traditional adult-centered perspectives often fail to capture (Zimmerman *et al*. 2004, Rose *et al*. 2025). This gap is particularly consequential for Hispanic adolescents, who navigate complex intersecting influences of developmental transitions, cultural identity, immigration experiences, family dynamics, and systemic inequities—all of which affect their mental health and access to care (Cubilla-Batista *et al*. 2017, Lightfoot *et al*. 2019) and whose voices are underrepresented in mental health research (Polanco-Roman and Miranda 2022).

The visual modality of Photovoice is particularly well-suited for adolescents who may struggle to verbalize complex emotional and cultural experiences but can often express them more freely through imagery (Roxas and Gabriel 2022). Despite its relevance, studies using Photovoice with Hispanic adolescents are scarce (Vaughn *et al*. 2008), contributing to the broader underrepresentation of their perspectives (Mawn *et al*. 2016, Stephens *et al*. 2023). These gaps highlight both a need and an opportunity to expand the use of Photovoice to center Hispanic adolescents’ voices and inform mental health interventions that are culturally and developmentally responsive.

### Current study

Conducted in partnership with two community youth centers as a part of a broader suicide prevention initiative to design a task-shifting approach to suicide prevention within these centers, this study explores two central questions: (i) How do adolescents conceptualize mental health? and (ii) What are their mental health priorities? Mental health priorities are defined as the key thematic areas, needs, and systemic or environmental factors that are most critical for achieving and maintaining adolescents’ mental well-being (Johns Hopkins Center for Global Mental Health 2025). This study contributes conceptually by using Photovoice to highlight the unique multilayered dynamics that impact Hispanic adolescents’ mental health, underscoring the need for prevention and intervention strategies at each level of the ecological system. Methodologically, it demonstrates that Photovoice is developmentally appropriate for adolescents, leveraging their creativity and making their lived experiences central to knowledge production (Stephens *et al*. 2023). In this study, we used the word Hispanic—instead of Latine or Latinx—to convey adolescent participants’ preferred term.

## Methods

The three main goals of Photovoice, as outlined by Wang and Burris (1997), are to empower participants: (i) to reflect on their realities, (ii) to engage in critical dialogue through group discussion of photographs, and (iii) to advocate/produce change. Our study focused primarily on reflection and dialogue. We conducted nine meetings [preparatory (*n* = 3), focus groups (*n* = 3), feedback (*n* = 3)] with 12 Hispanic adolescents aged 13–17 conducted from June 2024 to February 2025. A purposive sample of Hispanic adolescents was recruited from two youth community centers in Greater Boston that offer academic, vocational, and life skills strategies. Adolescents were identified and referred by youth program directors. Directors—who work closely with the adolescents—assessed adolescents’ availability to commit to participating in the study and meet eligibility criteria as follows: (i) enrolled in the youth community center, (ii) aged between 13 and 17, and (iii) self-identified as Hispanic/Latine/Latinx, including Afro-Hispanic/Latine/Latinx.

### Procedures

Program directors distributed English and Spanish study flyers to families and provided contact information for interested participants to the research team. Subsequently, a research assistant (RA) emailed parents introductory materials, including a video from the principal investigator (PI). The PI or RA then called parents to obtain verbal consent and schedule a formal enrollment meeting via phone or Zoom. Ahead of this meeting, the RA emailed consent and assent forms to the family. During the call, the research team reviewed these documents, answered questions, and obtained verbal consent from parents and assent from adolescents. Copies were then shared via email.

Adolescents received a digital camera to keep and were compensated $45 for completing three sequential meetings. Participants could choose to receive a lump sum after all sessions or $15 following each session. This study was approved by the Boston College Institutional Review Board (Protocol # 24.213).

### Ethical research with adolescents

To ensure the protection, autonomy, and well-being of adolescents in the study, the following ethical considerations were implemented: (i) Parental permission was required before approaching adolescent participants with information. In most US states, including Massachusetts, where the study took place, the legal age for research consent is 18. Individuals under this age are legally considered minors for research purposes under federal regulations (U.S. Department of Health and Human Services 2024). Thus, the university IRB required us to obtain parental consent and adolescent assent. (ii) Adolescents were informed that participation was voluntary. They were provided with clear information about time commitments, the audio recording of the session (the only session that was recorded), privacy protections, and the limitations of confidentiality. (iii) All study materials and procedures were tailored to be aligned with participants’ cognitive and emotional developmental stage. (iv) The researchers who facilitated the focus groups shared adolescents’ cultural background, which supported recognition and respect of cultural beliefs and values, particularly around topics such as mental health. Additionally, our broader research team was composed of individuals from diverse cultural and ethnic backgrounds. This diversity allowed us to critically reflect on and address potential biases or assumptions that might arise when researchers share cultural or linguistic backgrounds with participants. Through ongoing reflexivity and dialogue, we aimed to ensure that our interpretations remained grounded in the participants’ perspectives rather than our own. (v) We collaborated closely with directors and staff of youth community centers to build trust and ensure the research was culturally and contextually relevant. (vi) We prepared a plan for support and referral if any emotional or psychological needs emerged during the study. (vii) Finally, we acknowledge the inherent power differentials between adult researchers and adolescent participants. To minimize these through the research process, we selected Photovoice, a participatory method to center adolescents’ perspectives and give them control over what to photograph and share. During the meetings, the researchers facilitated rather than directed discussions, using accessible language and encouraging adolescents to share and lead these discussions. Additionally, by limiting meetings to three, we wanted to respect participants’ time and reduce burden, balancing methodological rigor with feasibility. These strategies aimed to foster mutual respect.

### Workshops and focus groups procedures

This Photovoice study engaged three distinct groups of adolescents in a series of workshops and focus groups designed to explore the central research question through participatory photography and dialogue. Each group participated in three sequential meetings, totaling nine sessions. Participants were divided into three separate cohorts based on their affiliation with the community centers. Two cohorts met at one community center and one at the other. Group sizes ranged from three to six participants (Nyumba *et al*. 2018). Each group met independently across three sessions, allowing for in-depth engagement and continuity within each cohort. Sessions lasted approximately 90 min. All sessions were led by the same team: the PI and the RA. Each session was led by the PI, while the RA took notes. The PI, the RA, and the participants are all bilingual (English and Spanish), and the sessions were conducted in English due to the participants’ preference.

#### 1. Preparatory workshop

In the first session, participants were introduced to the study’s purpose and central research questions. We utilized these questions as conceptual prompts, asking adolescents to discuss their definitions and interpretations (of conceptualization and priorities). As a group, it was decided to focus on the two questions: (i) defining mental health and (ii) reflecting on what was most important to their mental health and well-being. The last question served as a roadmap to understand points of potential interventions and supports and allowed us to develop the aforementioned task-shifting project. Researchers provided an overview of the Photovoice methodology, emphasizing its role in empowering youth to document, reflect on their lived experiences, and engage in critical dialogue through photography. Participants received training on the ethical use of digital cameras, including safety (prioritizing adolescents’ safety over getting the photograph), principles of respect for persons, guidance on informed consent, and instruction on explaining the study's goals to potential subjects before photographing them. At the end of the meeting, it was agreed that adolescents would not take photos of people's faces for respect of their privacy (Wang and Redwood-Jones 2001). Adolescents were given 1 or 2 weeks to take photos before the photo-sharing meeting. Following methodological Photovoice recommendations (Nykiforuk *et al*. 2011), we encouraged adolescents to focus on both research questions and select one or two photographs to discuss in the next focus group.

#### 2. Focus group

In the second session, participants returned with photographs they had taken in response to the research prompts. They were asked to prioritize two photographs to share with the group, and the session was audio-recorded. Guided by the SHOWeD technique—a structured framework for critical reflection—participants engaged in group dialogue to interpret and contextualize their images (Wang and Burris 1997). The SHOWeD questions included the following: S: What do you *See* here? H: What is really *Happening*? O: How does this relate to *Our* lives? W: *Why* does this situation, concern, or strength exist? D: What can we *Do* about it? This process facilitated peer learning, collective meaning-making, and the identification of topics grounded in participants lived experiences. The audio recordings from this session were transcribed by a bilingual (English and Spanish) person outside the research team who has extensive experience transcribing qualitative interviews.

#### 3. Feedback workshop

Although not part of Braun and Clarke's analytical approach to reflexive thematic analysis, this meeting allowed researchers to present a synthesized interpretation of the data, drawing on quotes from the audio-recorded focus group. Participants were invited to confirm, refine, or challenge these interpretations, ensuring that the findings accurately reflected adolescents’ visual and narrative data. This session also provided an opportunity to discuss next steps, including potential avenues for dissemination and provided evidence for a task-shifting approach to suicide prevention in their youth community centers. Providing captions to photographs is commonly done in Photovoice; however, we did not ask adolescents to title their photos for this study. We are planning to organize a photograph exhibition and adolescents will have the opportunity to caption their photographs

### Data sources

In our analysis, we incorporated textual transcripts, notes, and participant-generated photographs. The visual data (photographs) were primarily used during the focus group as prompts to elicit adolescents’ interpretations of their images, reflecting on their realities, communities, and cultural contexts. While our analysis systematically analyzed textual data and notes, we did not incorporate both textual and visual data together in a systematic analytical process (e.g. textual-visual thematic analysis; Trombeta and Cox 2022). Although such an approach is recommended, rigorously implementing it in a participatory way would have required additional group sessions beyond the three meetings, potentially creating a significant burden for participants (Murray and Nash 2016). Therefore, we deliberately prioritized adolescents’ time and engagement by balancing the analysis with feasibility within the Photovoice project.

### Data analysis

Four team members engaged in reflexive thematic analysis (Braun and Clarke 2019, 2024) using audio recordings of focus groups, notes taken during all sessions, and adolescents’ feedback. The analysis was led by the PI alongside one PhD- and two master's-level students. Analysts identified as Latinas and Afro-Caribbean and shared language, cultural, and experiential backgrounds with the participants. The reflexive approach to thematic analysis highlights the role of researchers in knowledge production (Braun and Clarke 2024). We acknowledge that knowledge is socially constructed and that our cultural backgrounds, our training as social workers and counselors, and theoretical assumptions about Hispanic adolescents in the USA, intersect with this study's data. An inductive approach was adopted, meaning data were open-coded; however, a degree of deductive analysis was used to ensure the open codes were meaningful to the research questions. A six-phase analytical process (Braun and Clarke 2019) facilitated the analysis. First, the research team familiarized itself with the data by reviewing notes, listening to the audio recordings, and reading the transcripts. Initial codes were generated collaboratively using Excel to organize and sort the data. Codes were developed inductively, grounded in the participants’ language and perspectives, and refined through iterative discussion among the research team and feedback from the participants. Mental health conceptualization and priorities were identified through a process of constant comparison across focus groups, with attention to both shared and divergent meanings. Because the analysis occurred concurrently with data collection, the team was able to reflect on discussed topics in real time and adapt subsequent discussions to explore these ideas more deeply. Data from the focus groups along with notes and participant feedback provided sufficient information power to develop a robust and nuanced analysis of mental health conceptualization and priorities (Braun and Clarke 2021). This iterative process enhanced the depth and relevance of the findings (Rudkin and Davis 2007). Throughout the analysis, we engaged in reflexive dialogue to consider how our own cultural backgrounds and positionalities might shape interpretation (Braun and Clarke 2019). The diversity of the research team and adolescents’ feedback facilitated a critical examination of assumptions and ensured that the topics remained grounded in adolescents’ lived experiences.

## Results

**Figure 1:**
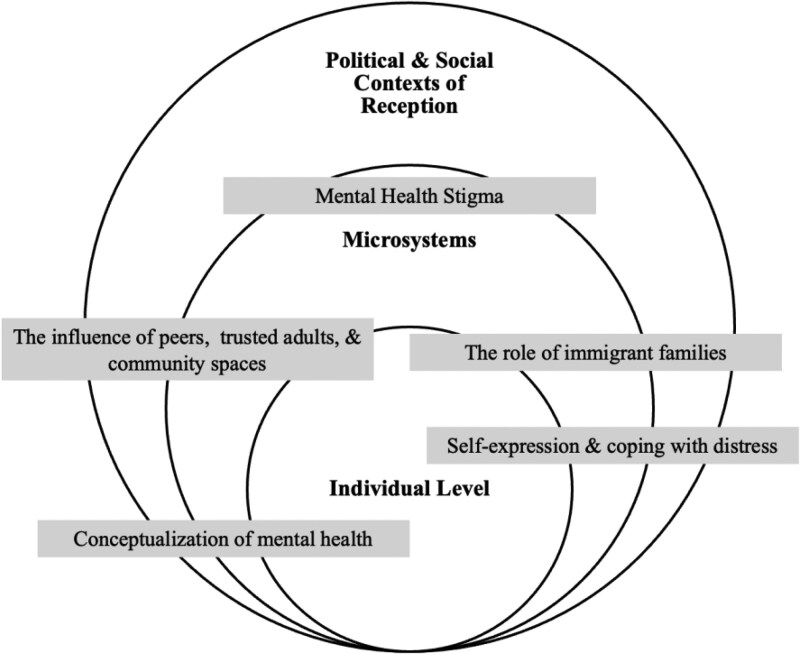
Hispanic adolescent perceptions mapped onto the framework (based on Suarez-Orozco *et al.* 2018). Source: Developed by authors, based on the conceptual model by Suarez-Orozco *et al.* (2018).

Table 1 provides background characteristics of twelve adolescent participants (ages 13–17) who conceptualized mental health not as a static, but as a dynamic process shaped by multiple ecological contexts. Their definition mirrored the Suarez-Orozco *et al.* (2018) model, emphasizing that development is “complex and unique” inextricably linked to interconnected individual, familial, cultural, and environmental factors (Fig. 1).

**Table 1:** Participant characteristics.

		Youth community center	Age	Sex	Ethnic identity
1	Gloria	1	17	Female	Hispanic
2	Ken	1	17	Male	Hispanic
3	Jim	1	14	Male	Hispanic
4	Roy	1	15	Male	Hispanic-Black
5	Nina	1	15	Female	Hispanic
6	Dan	1	15	Male	Hispanic
7	Ema	1	15	Female	Hispanic
8	Ismael	2	15	Male	Hispanic
9	Carmen	2	16	Female	Hispanic
10	Mario	2	15	Male	Hispanic-Black
11	Lala	2	13	Female	Hispanic
12	Sebastian	2	14	Male	Hispanic-Black

All adolescents were bilingual (English and Spanish). English was their preferred language for the meetings.

### Adolescent conceptualization of mental health

**Figure 2:**
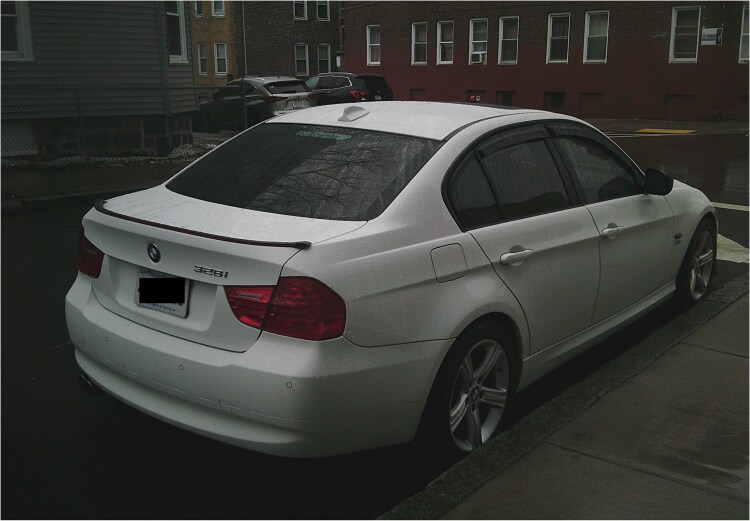
The car as a metaphor for mental health.

Adolescents viewed mental health as multifaceted internal-external system, illustrating the interdependence of developmental domains and context. Jim’s metaphor of the car (Fig. 2) noted that “your whole body has parts like a car, and while they can be ‘repaired,’ systemic ‘crashes’ occur when the ‘direction (contextual path) is wrong,’ and everything that happened leading up to right now…all the things you have been taking for long.” Adolescents’ discussions also suggested an intuitive understanding of cumulative risk and its impact on mental health as illustrated by this conversation.

Jim: It’s also just how you grew up in general. So, like, everything that happened leading up to right now.

Roy: Like, all the things you’ve been taking for long.

Dan: Like childhood trauma.

The complexity of mental health was further interpreted through metaphors of invisibility, such as Nina's “glacier” and Roy's “car exterior colors” explaining “we don’t see the bottom of a car that’s, like, a person, we don’t see, like, the inside.” These descriptions move beyond simple definitions of “sadness” and instead highlight a sophisticated awareness of internalizing symptoms, the “hidden” layers of the self that are often obscured by external behaviors or people's perceptions.

Signs of emotional distress were frequently framed as a conflict between internal experience and external perception. Participants identified withdrawal and “crying in secret” (Emma, 16) as responses to a perceived lack of safety in their immediate microsystems (e.g. home). As one participant noted, what adults perceive as “catching an attitude” is often a manifestation of underlying distress, suggesting a breakdown in cross-contextual communication between adolescents and their primary caregivers.

### Adolescent mental health priorities

Adolescent priorities were centered on the tension between individual coping and the sociocultural pressures inherent in immigrant-origin households.

#### Self-expression and coping with distress

**Figure 3:**
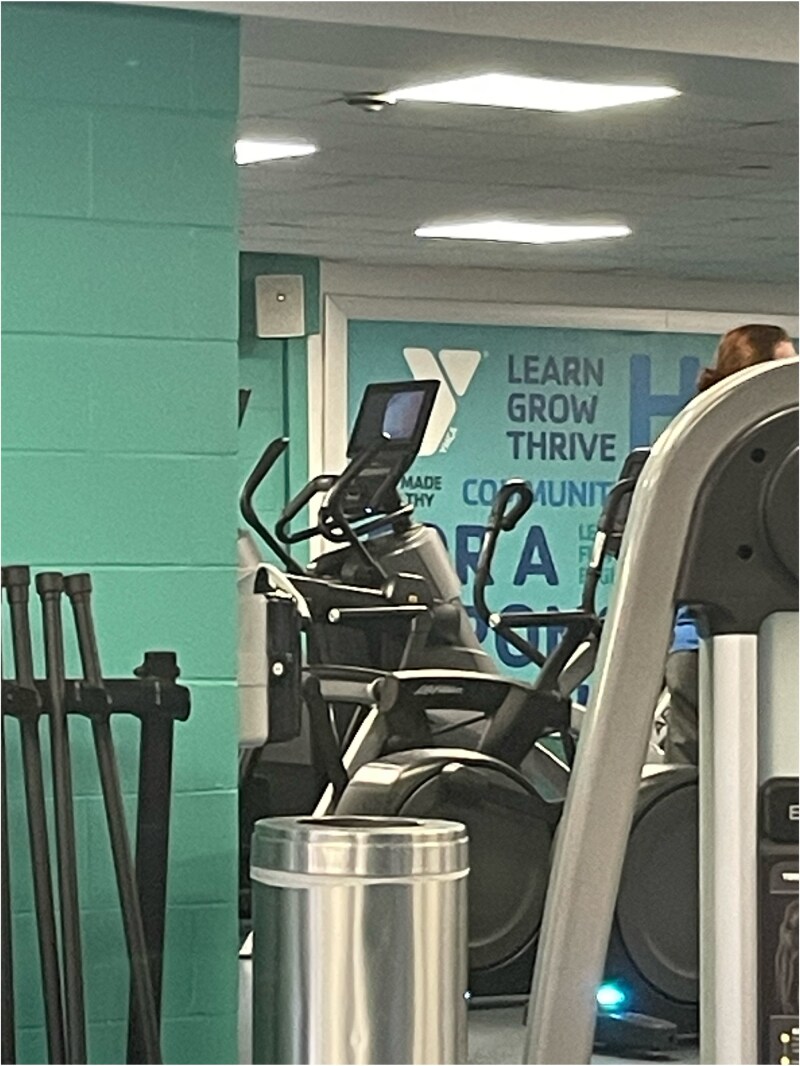
The gym with inspiring notes.

Coping was identified as a primary resilience factor. Participants utilized creative and kinetic strategies (e.g. fashion, exercise, photography) to reclaim agency. Ema's focus on her sneakers as a “happy part of mental health” represents the use of material culture for identity signaling and self-regulation. Similarly, utilizing neighborhood “green spaces” or community centers (the gym) demonstrates how the built environment serves as a vital resource for “letting anger out” and achieving “peace.” For example, with a photo of a gym (Fig. 3), Sebastian, a 14-year-old, Black Latino noted:

….when I am mad and I get my anger out in the gym.

#### The role of immigrant families in adolescent mental health

Living within immigrant families was a common experience among the adolescents in the study. The vicissitude of the immigrant family was a significant contextual risk factor, specifically regarding intergenerational roles and responsibilities. Ken's reflection on being a 6-year “translator” highlights the acculturative stress and premature autonomy often entrusted upon immigrant-origin adolescents. He said:

Here in our community, most come from immigrant families…a lot of teenagers feel like they have pressure on them, because they’re the first kids coming from an immigrant household. I come from a single-parent home, immigrant parents, which is kind of hard for… because at a young age, they want you to translate like paperwork and stuff like that, and, you know. What type of six-year-old is going to be able to translate that whole constitution?

Furthermore, the data revealed a developmental mismatch between adolescents’ needs and parental “traditionalist” attitudes. This was framed not as a lack of love, but a byproduct of the parents’ own cultural and sociopolitical history. Roy and Nina interpreted their parents’ denial of mental health struggles as a survival mechanism—a “bloodline” of suppression shaped by historical traumas like the Trujillo dictatorship. The following quote from Nina illustrates the chronosystemic influence (Suarez-Orozco *et al*. 2018), where past political contexts in the country of origin continue to shape parenting styles and emotional validation in the host country.

I feel like since I’m Dominican, all Dominicans were shaped by Trujillo. And, like, they saw how Trujillo treated their parents. And so, their parents got shaped into something. And so, they brought it on to us…they don't like to spend [time] or talk. They want to be strong.

Finally, some adolescents noted a gap in the mesosystem where familial expectations, perceptions of mental health, and gender socialization (often rooted in the parents’ country of origin) clash with the adolescents’ lived experience leading to feelings of being misunderstood or unseen regarding their mental health. The following quote by Emma illustrates this cultural dissonance:

We’re being told that…well, in a Hispanic household, that you belong to the kitchen. And that you can’t, like, you have to marry a man just for the kitchen and to take care of the kids. But you’re also told as a female that you can’t cry. Because you have no reason to be crying…and parents don’t really think that we have anything to stress about or feel depressed because they were raised a certain way back in their days.

#### The role of peers, trusted adults, and the community in adolescent mental health

Adolescents discussed the importance of immediate settings (microsystems such as home, school and community centers) and the relationships within these systems. Ismael and Carmen described school and community centers as critical microsystems. School was seen as a “social environment” to find common ground and support. However, it was also seen as a source of stress, visible in Carmen's note that school is also a source of “high expectations.” Despite their desire to talk to someone in school and awareness of mental health providers in school, Nina and Dan highlighted a failure in these important systems of support. While schools provide counselors, the adolescents felt these adults are “forced to listen” or “paid to listen,” eroding relational trust which is essential for successful development.

Within the home, some adolescents felt they could not communicate with their parents, in part because they did not want to add additional burden. When home and school failed youth, community centers act as a vital third space. Mentors in these centers provide the engaged body language and authentic connection necessary for resilience. This is illustrated in the following quote by Ken, who grew up in a single-parent household:

I usually trust people by their body language, it shows if they’re actually engaged or not. Or if they’re acting like they are, but they’re not. And that’s what I honestly got from him [mentor at the youth community center]. He’s very engaged with the youth. And just him, like, being up there talking to everybody about how mental health is important.

#### Mental health stigma

Adolescent participants described cultural stigma surrounding mental health. Ken and Nina's observations about mental health being viewed as a “sickness” or “only experienced by crazy people” reflect macrosystemic beliefs common in many immigrant cultures. Additionally, gender socialization and its impact on mental health expression are clearly supported by Roy's quote indicating how this cultural blueprint dictates how emotions are managed, leading to the “piling up” of trauma.

…For men, in a Hispanic household, you’d be like, oh, men don’t cry. You always hear that. And I think, usually men, we, they just keep that. Like, we just keep it and don’t tell people. It’s what we’re told…So they just stay silent and just move on. Just creating that trauma, piling up. Until one day, when they see they cannot keep on going.

Nina's comment about “the whole bloodline” showing grandparents, parents, and adolescents to “suck it up” illustrates how cultural values about mental health are passed down through generations, often creating a dissonance between the mental health needs of adolescents. Therefore, these immigrant-origin adolescent participants often internalized distress affecting their socio-emotional development as illustrated in the following quote by Gloria:

But most of the time, it’s like, you bottled up so much, to the point where you just break … there’s a point … in my life where I was bottling up all my emotions so badly that I would just cry myself to sleep, because I felt like I couldn’t talk about it.

Adolescents suggested that parents “need to be more educated” to reduce stigma about mental health and increase communication of emotional distress, aligning with a call for interventions that help parents become “cultural translators” who can understand the new stressors that adolescents face (Suarez-Orozco *et al*. 2018).

## Discussion

This Photovoice study examined how Hispanic adolescents conceptualized mental health, revealing a multidimensional, culturally embedded understanding shaped by personal experiences, immigrant family dynamics, cultural identity, and community context. Through photographs and reflective dialogue, adolescent participants defined mental health as a dynamic, individualized experience that transcends clinical definitions. They identified signs of distress, such as sadness, withdrawal, isolation, and fear of judgment—symptoms often associated with mental health conditions (American Psychiatric Association 2013). This awareness reflects not only their emotional insight but also their ability to articulate these experiences. Their narratives demonstrated a nuanced awareness of the emotional complexity of adolescence, particularly for immigrant-origin adolescents. These findings align with and support the integrative model for the adaptation of immigrant-origin children and youth (Suárez-Orozco *et al*. 2018). This model posits that an adolescent’s successful development and mental health hinge on a dual process: achieving developmental and psychological milestones (e.g. self-regulation, academic progress, self-esteem) while simultaneously acquiring the cultural competencies necessary to effectively navigate the two distinct worlds of home and the wider community. This study extends the literature on Hispanic mental health by centering the adolescents’ visual and narrative voices, a feature largely absent in prior research. We move beyond documenting disparities to illuminate the lived experiences of structural and mental health challenges through participants’ own perspectives.

Adolescents employed metaphors, such as cars, glaciers, and roller coasters, to convey the layered and dynamic nature of mental health. The metaphor of the car, for instance, illustrated the interconnectedness of emotional and physical well-being, emphasizes the need for ongoing care. This aligns with prior research indicating that adolescents often use tangible analogies to make sense of abstract emotional experiences (Stephens *et al*. 2023), which can inform mental health literacy and interventions. The use of metaphors also underscores the value of Photovoice as a method, allowing adolescents to communicate thoughts that might otherwise remain unspoken (Vélez-Grau 2019). This approach not only facilitates self-reflection but also empowers adolescents to define mental health on their own terms, which may reduce isolation and stigma. Culturally grounded interventions should incorporate metaphors, storytelling, and creative expression to resonate with adolescents’ lived experiences.

Consistent with previous Photovoice studies exploring mental health among adolescents (Stephens *et al*. 2023), participants prioritized diverse coping mechanisms—solitary, sensory, creative, and social. Activities such as walking, exercising, cleaning, listening to music, and engaging in self-care were described as mechanisms for emotional regulation. Creative outlets like fashion, murals, and photography served as tools for identity expression and emotional processing. These strategies reflect adolescents’ pursuit of autonomy and self-definition, underscoring the therapeutic and empowering potential of accessible, everyday practices in mental health and well-being. Such approaches offer meaningful avenues for agency and resilience amid stress, stigma, and sociocultural complexity.

As proposed in the integrative model (Suárez-Orozco *et al*. 2018), adolescents’ mental health experiences were deeply shaped by life within immigrant families. Participants described navigating bicultural identities—speaking Spanish at home and English in outside environments—highlighting the tensions and resilience embedded in this dual existence. Many assumed adult responsibilities early, experienced cultural dissonance, and felt emotionally invalidated by parents due to generational and sociopolitical divides. These dynamics, consistent with prior research on Latino/Hispanic families (Zayas 2011, Calzada *et al*. 2013, Fortuna *et al*. 2016, Vélez-Grau *et al*. 2023), may contribute to internalized pressure and emotional suppression, particularly in households where traditional values discourage emotional vulnerability. Participants noted that their parents likely faced similar adolescent struggles but were socialized to conceal them, perpetuating intergenerational silence and emotional invisibility, aspects consistent with the intergenerational conflicts and goal misalignments described in the integrative model for the adaptation of immigrant-origin children and youth (Suarez-Orozco *et al*. 2018). These findings underscore the need for culturally responsive, family-based interventions that promote open communication. Strategies may reinforce adolescents’ use of parents’ native language to enhance emotional connection with monolingual parents (Tseng and Fuligni 2000, Suárez-Orozco *et al*. 2018, Vélez-Grau *et al*. 2023, Cox Jr *et al*. 2025).

Trusted adults or mentors were central to adolescents’ vision of mental health support. Mentoring, an evidence-based practice, supports adolescents and improves mental health outcomes, serving both as a prevention and intervention strategy (Fabbi 2021). Because mentors often share cultural backgrounds and lived experiences, they can be trained to deliver culturally relevant mental health interventions (Wyman *et al*. 2010, King *et al*. 2019). This supports the promise of task-shifting—training non-specialists—to expand access, build trust, and aligned services with adolescents’ needs (Vélez-Grau and Alvarez 2024). We recommend that community-based supports, including youth centers, mentorship programs, and peer networks, be expanded to provide safe, affirming environments.

Finally, stigma was identified as a pervasive barrier. Participants feared being labeled “sensitive” or “crazy,” echoing findings that Latino/a adolescents report higher stigma than non-Latino White peers (DuPont-Reyes *et al*. 2020). This stigma often led to emotional suppression and isolation. Gendered expectations—such as “men don't cry” or “girls have nothing to worry about”—further discouraged help-seeking (Lindsey *et al*. 2017). Participant adolescents called for parental education on mental health to break cycles of suppression and promote emotional validation. These findings support calls for culturally tailored mental health literacy and stigma-reduction interventions to address mental health disparities among the Hispanic population (Pérez-Flores and Cabassa 2021). Collectively, these findings underscore the importance of adolescent-centered, culturally responsive, and multisystemic approaches when working with Hispanic adolescents (Ramirez *et al*. 2021). As such, this study findings provided evidence for a task-shifting approach to suicide prevention tailored for Hispanic adolescents in youth community centers, which aligns with the third goal of Photovoice: taking action. For future research, this study underscores the need to broaden the scope of knowledge production by continuing to leverage visual participatory methods to co-create, implement, and evaluate multisystemic and culturally responsive interventions (involving family, school, and community) that are designed to advance mental health equity in immigrant-origin communities.

The present study has a few limitations worth considering. First, the participants were recruited through youth centers in Greater Boston. Their experiences may differ from those of adolescents who are more isolated and may not have access to these resources. All participants identified as Hispanic; however, the present sample does not capture the wide range of diversity of this population (e.g. country of origin, socioeconomic status, immigration status). Only the second session was audio-recorded, which may have omitted important information from sessions one and three, and could have increased reliance on notes from these sessions. Additionally, visual data was not analyzed in conjunction with textual data to avoid burdening adolescents with time spent in the project. Despite these limitations, the strength of this study lies in its utilization of Photovoice; this methodology successfully generated nuanced, participant-driven insights into mental health conceptualizations that transcend the limitations of traditional, quantitative methods (Han and Oliffe 2016).

## Conclusion

This Photovoice study extends the literature on Hispanic adolescent mental health by centering their visual and narrative voices. The findings indicated that mental health cannot be confined to one space; it is multilayered and multisystemic. Mental health for Hispanic adolescents is shaped by culture, context, and sociopolitical forces. The adolescents’ priorities—safe spaces, creative outlets, and open dialogue—provide a clear roadmap for action. We conclude that culturally responsive and multisystemic interventions involving family, school, and community settings are essential to validate lived experiences, dismantle stigma, and advance mental health equity for this population. Future research must continue to leverage participatory methods like Photovoice to co-create these adolescent-led solutions.

## Data Availability

Due to ethical and privacy considerations, including identifiable photographs and sensitive qualitative discussions, the data from this Photovoice study are not publicly available. De-identified excerpts may be provided upon reasonable request to the corresponding author.

## References

[daag032-B1] Alegria M, Carson NJ, Goncalves M et al Disparities in treatment for substance use disorders and co-occurring disorders for ethnic/racial minority youth. J Am Acad Child Adolesc Psychiatry 2011;50:22–31. 10.1016/j.jaac.2010.10.005 21156267 PMC3488852

[daag032-B2] American Psychiatric Association . Diagnostic and Statistical Manual of Mental Disorders. 5th ed. Washington, DC: American Psychiatric Association, 2013. 10.1176/appi.books.9780890425596

[daag032-B3] Braun V, Clarke V. Reflecting on reflexive thematic analysis. Qual Res Sport Exerc Health 2019;11:589–97. 10.1080/2159676X.2019.1628806

[daag032-B4] Braun V, Clarke V. To saturate or not to saturate? Questioning data saturation as a useful concept for thematic analysis and sample-size rationales. Qual Res Sport Exerc Health 2021;13:201–16. 10.1080/2159676X.2019.1704846

[daag032-B5] Braun V, Clarke V. A critical review of the reporting of reflexive thematic analysis in in Health Promotion International. Health Promot Int 2024;39:daae049. 10.1093/heapro/daae049 38805676 PMC11132294

[daag032-B6] Calzada EJ, Tamis-LeMonda CS, Yoshikawa H. Familismo in Mexican and Dominican families from low-income, urban communities. J Fam Issues 2013;34:1696–724. 10.1177/0192513X12460218

[daag032-B7] Centers for disease Control and Prevention . *Youth Risk Behavior Survey data Summary and Trends Report: 2013–2023*. U.S. Department of Health and Human Services. 2024. https://www.cdc.gov/yrbs/dstr/index.html (25 September 2025, date last accessed)

[daag032-B8] Cox RB, Arredondo-Lopez A, León-Cartagena M et al Families navigating two languages in a country built for one: shared language erosion among Hispanic parents and youth. J Soc Pers Relat 2025;42:1849–79. 10.1177/02654075251332034

[daag032-B9] Cubilla-Batista I, Andrade EL, Cleary SD et al Picturing Adelante: Latino youth participate in CBPR using place-based photovoice. Soc Mar Q 2017;23:18–35. 10.1177/1524500416656586

[daag032-B10] DuPont-Reyes MJ, Villatoro AP, Phelan JC et al Adolescent views of mental illness stigma: an intersectional lens. Am J Orthopsychiatry 2020;90:201–11. 10.1037/ort0000425 31380669 PMC7000296

[daag032-B11] Fabbi C . Mentoring and Mental Health. Boston, MA: MENTOR: The National Mentoring Partnership, 2021. https://www.mentoring.org/

[daag032-B12] Fortuna LR, Alvarez K, Ortiz ZR et al Mental health, migration stressors and suicidal ideation among Latino immigrants in Spain and the United States. Eur Psychiatry 2016;36:15–22. 10.1016/j.eurpsy.2016.03.001 27311103 PMC5500916

[daag032-B13] Grieb SM, Platt R, Vazquez MG et al Mental health stigma among Spanish-speaking Latinos in Baltimore, Maryland. J Immigr Minor Health 2023;25:999–1007. 10.1007/s10903-023-01488-z 37213041 PMC10201042

[daag032-B14] Han CS, Oliffe JL. Photovoice in mental illness research: a review and recommendations. Health 2016;20:110–26. 10.1177/1363459314567790 25673051 PMC4768711

[daag032-B15] Ijadi-Maghsoodi R, Bonnet K, Feller S et al Voices from minority youth on help-seeking and barriers to mental health services: partnering with school-based health centers. Ethn Dis 2018;28:437–44. 10.18865/ed.28.S2.437 30202197 PMC6128338

[daag032-B16] Johns Hopkins Center for Global Mental Health . *Youth Mental Health Spotlighted at U.S*. *Launch of the Second Lancet Commission on Adolescent Health and Wellbeing*. 2025. Retrieved December 16 from https://publichealth.jhu.edu/center-for-global-mental-health/youth-mental-health-spotlighted-at-us-launch-of-the-second-lancet-commission-on-adolescent-health-and-wellbeing (17 December 2025, date last accessed)

[daag032-B17] King CA, Arango A, Kramer A et al Association of the Youth-Nominated Support Team intervention for suicidal adolescents with 11- to 14-year mortality outcomes: secondary analysis of a randomized clinical trial. JAMA Psychiatry 2019;76:492–8. 10.1001/jamapsychiatry.2018.4358 30725077 PMC6495350

[daag032-B18] Leal CC . Stigmatization of Hispanic children, pre-adolescents, and adolescents with mental illness: exploration using a national database. Issues Ment Health Nurs 2005;26:1025–41. 10.1080/01612840500280695 16283997

[daag032-B19] Lightfoot AF, Thatcher K, Simán FM et al What I wish my doctor knew about my life”: using photovoice with immigrant Latino adolescents to explore barriers to healthcare. Qual Soc Work 2019;18:60–80. 10.1177/1473325017704034 32973399 PMC7510170

[daag032-B20] Lindsey MA, Brown DR, Cunningham M. Boys do (n’t) cry: addressing the unmet mental health needs of African American boys. Am J Orthopsychiatry 2017;87:377–83. 10.1037/ort0000198 28691838

[daag032-B21] Martinez AR . Intersectionality, voz, and agency: a culture-centered approach to understanding US-born Mexican Americans’ depression experiences. South Commun J 2017;82:278–97. 10.1080/1041794X.2017.1347702

[daag032-B22] Mawn L, Welsh P, Kirkpatrick L et al Getting it right! Enhancing youth involvement in mental health research. Health Expect 2016;19:908–19. 10.1111/hex.12386 26202658 PMC5152725

[daag032-B23] Merikangas KR, He J-p, Burstein M et al Lifetime prevalence of mental disorders in US adolescents: results from the National Comorbidity Survey Replication–Adolescent Supplement (NCS-A). J Am Acad Child Adolesc Psychiatry 2010;49:980–9. 10.1016/j.jaac.2010.05.017 20855043 PMC2946114

[daag032-B24] Mikolajczyk RT, Bredehorst M, Khelaifat N et al Correlates of depressive symptoms among Latino and non-Latino White adolescents: Findings from the 2003 California Health Interview Survey. BMC Public Health 2007;7:21–9. 10.1186/1471-2458-7-21 17313675 PMC1805430

[daag032-B25] Murray L, Nash M. The challenges of participant photography: a critical reflection on methodology and ethics in two cultural contexts. Qual Health Res 2016;27:923–37. 10.1177/1049732316668819 27634295

[daag032-B26] Nykiforuk CI, Vallianatos H, Nieuwendyk LM. Photovoice as a method for revealing community perceptions of the built and social environment. Int J Qual Methods 2011;10:103–24. 10.1177/160940691101000201 27390573 PMC4933584

[daag032-B27] Nyumba TO, Wilson K, Derrick CJ et al The use of focus group discussion methodology: insights from two decades of application in conservation. Methods Ecol Evol 2018;9:20–32. 10.1111/2041-210X.12860

[daag032-B28] Pérez-Flores NJ, Cabassa LJ. Effectiveness of mental health literacy and stigma interventions for Latino/a adults in the United States: a systematic review. Stigma Health 2021;6:430–9. 10.1037/sah0000343 35368243 PMC8974450

[daag032-B29] Polanco-Roman L, Miranda R. A cycle of exclusion that impedes suicide research among racial and ethnic minority youth. Suicide Life Threat Behav 2022;52:171–4. 10.1111/sltb.12752 33811663 PMC10438926

[daag032-B30] Polo AJ, Solano-Martinez JE, Saldana L et al The epidemic of internalizing problems among Latinx adolescents before and during the Coronavirus 2019 pandemic. J Clin Child Adolesc Psychol 2024;53:66–82. 10.1080/15374416.2023.2169925 36998122

[daag032-B31] Ramirez AG, Lepe R, Cigarroa F. Uplifting the Latino population from obscurity to the forefront of health care, public health intervention, and societal presence. JAMA 2021;326:597–8. 10.1001/jama.2021.11997 34402821

[daag032-B32] Roche KM, Huebner DM, Lambert SF et al COVID-19 stressors and Latinx adolescents’ mental health symptomology and school performance: a prospective study. J Youth Adolesc 2022;51:1031–47. 10.1007/s10964-022-01603-7 35381907 PMC8983080

[daag032-B33] Rose T, Leitch J, Forrester P et al “Everybody’s mind is different. Like, it’s not gonna work the same”: an exploratory study of youth perspectives on mental health. Youth Soc 2025;57:982–1008. 10.1177/0044118X241296553

[daag032-B34] Roxas K, Gabriel ML. Research that we need: re-centring of immigrant youth and their families through photovoice projects. Intercult Educ 2022;33:35–47. 10.1080/14675986.2021.2017594

[daag032-B35] Rudkin JK, Davis A. Photography as a tool for understanding youth connections to their neighborhood. Child Youth Environ 2007;17:107–23. 10.1353/cye.2007.0000

[daag032-B36] Sanchez D, Chavez FLC, Capielo Rosario C et al Racial differences in discrimination, coping strategies, and mental health among US Latinx adolescents during COVID-19. J Clin Child Adolesc Psychol 2024;53:114–28. 10.1080/15374416.2024.2301762 38270572

[daag032-B37] Stephens M, Keiller E, Conneely M et al A systematic scoping review of photovoice within mental health research involving adolescents. Int J Adolesc Youth 2023;28:1–26. 10.1080/02673843.2023.2244043

[daag032-B38] Suárez-Orozco C, Motti-Stefanidi F, Marks A et al An integrative risk and resilience model for understanding the adaptation of immigrant-origin children and youth. Am Psychol 2018;73:781–96. 10.1037/amp0000265 30188166

[daag032-B39] Tran JT, Tran AX. Culturally responsive civic engagement: a pathway to mental health equity for Latinx youth. HCA Healthc J Med 2022;3:311–6. 10.36518/2689-0216.1427 37425255 PMC10327946

[daag032-B40] Trombeta G, Cox SM. The textual-visual thematic analysis: a framework to analyze the conjunction and interaction of visual and textual data. Qual Rep 2022;27:1557–74. 10.46743/2160-3715/2022.5456

[daag032-B41] Tseng V, Fuligni AJ. Parent-adolescent language use and relationships among immigrant families with East Asian, Filipino, and Latin American backgrounds. J Marriage Fam 2000;62:465–76. 10.1111/j.1741-3737.2000.00465.x

[daag032-B42] U.S. Department of Health and Human Services . *Subpart D—Additional Protections for Children Involved as Subjects in Research*. 2024. https://www.hhs.gov/ohrp/regulations-and-policy/regulations/45-cfr-46/common-rule-subpart-d/index.html#46.402 10259817

[daag032-B43] Vaughn LM, Rojas-Guyler L, Howell B. Picturing” health: a photovoice pilot of Latina girls’ perceptions of health. Fam Community Health 2008;31:305–16. 10.1097/01.FCH.0000336093.39066.e9 18794637

[daag032-B44] Vélez-Grau C . Using photovoice to examine adolescents’ experiences receiving mental health services in the United States. Health Promot Int 2019;34:912–20. 10.1093/heapro/day043 29986026

[daag032-B45] Vélez-Grau C, Alvarez K. Looking at global mental health models to prevent youth suicide in the United States. J Adolesc Health 2024;75:700–2. 10.1016/j.jadohealth.2024.07.022 39269380 PMC11536360

[daag032-B46] Vélez-Grau C, Magan IM, Gwadz M. The burden of not belonging: a qualitative study of the applicability of the interpersonal theory of suicide constructs of belongingness and burdensomeness to ethnocultural minoritized youth. Behav Ther 2023;54:777–93. 10.1016/j.beth.2023.02.004 37597957

[daag032-B47] Vélez-Grau C, Mufson L, Buelvas K et al A qualitative study of the impact of COVID-19 on family relationships and the lives of Latinx adolescents. J Child Fam Stud 2024;33:3093–103. 10.1007/s10826-024-02919-7

[daag032-B48] Verlenden JV, Fodeman A, Wilkins N et al Mental health and suicide risk among high school students and protective factors—Youth Risk Behavior Survey, United States, 2023. MMWR Suppl 2024;73:79–86. 10.15585/mmwr.su7304a9 39378246 PMC11559681

[daag032-B49] Wang C, Burris MA. Photovoice: concept, methodology, and use for participatory needs assessment. Health Educ Behav 1997;24:369–87. 10.1177/109019819702400309 9158980

[daag032-B50] Wang CC, Redwood-Jones YA. Photovoice ethics: perspectives from Flint photovoice. Health Educ Behav 2001;28:560–72. 10.1177/109019810102800504 11575686

[daag032-B51] Wyman PA, Cross W, Brown H et al Intervention to strengthen emotional self-regulation in children with emerging mental health problems: proximal impact on school behavior. J Abnorm Child Psychol 2010;38:707–20. 10.1007/s10802-010-9398-x 20180009 PMC2880630

[daag032-B52] Yonas MA, Burke JG, Miller E. Visual voices: a participatory method for engaging adolescents in research and knowledge transfer. Clin Transl Sci 2013;6:72–7. 10.1111/cts.12028 23399093 PMC3575688

[daag032-B53] Zaff JF, Aasland K, McDermott E et al Exploring positive youth development among young people who leave school without graduating high school: a focus on social and emotional competencies. Qualitative Psychology 2016;3:26–45. 10.1037/qup0000044

[daag032-B54] Zayas LH . Latinas Attempting Suicide: When Cultures, Families, and Daughters Collide. New York, NY: Oxford University Press, 2011 April 22.

[daag032-B55] Zimmerman MA, Morrel-Samuels S, Wong N et al Guns, gangs, and gossip. J Early Adolesc 2004;24:385–411. 10.1177/0272431604268551

